# Assessing the Use of BotulinumtoxinA for Hyperactive Urinary Tract Dysfunction a Decade After Approval

**DOI:** 10.3390/toxins17070351

**Published:** 2025-07-13

**Authors:** Heinrich Schulte-Baukloh

**Affiliations:** 1 Department of Urology, Charité—University Hospital, 12203 Berlin, Germany; heinrich.schulte-baukloh@charite.de; 2 Urologic Practice Turmstrasse, 10551 Berlin, Germany; 3 Department of Urology, University Hospital Brandenburg, 14770 Brandenburg, Germany

Botulinum toxin was approved by the Food and Drug Administration in 2011 for neurogenic bladder and in 2013 for idiopathic overactive bladder (OAB). In this Special Issue, we address the question of where we stand with this therapy a decade later. Has it prevailed and been proven useful? Which techniques have become established, and how were they optimized? Are more practical ways of distribution on the way? What acceptance problems or obstacles to long-term use might exist among patients or doctors? What are the risks? Has the therapy gained a foothold in the outpatient sector?

We are pleased to present you with diverse, largely practical aspects of botulinum toxin-A detrusor injections (BoNT/A-DI).

A review by E. Enemchukwu et al. (1) summarizes interesting aspects of various injection techniques from 43 seminal articles. Ongoing debate exists on whether 20 injections are necessary (as recommended in the AbbVie package insert) or fewer would achieve the same results. Based on this work, evidence suggests that fewer injections (comparing 1–10 vs. 20–40) maintain efficacy while reducing treatment time and the associated discomfort and pain. Various injection sites in the bladder lead to different levels of effectiveness, seemingly higher for detrusor (although this may result in an increased risk of urinary retention) than suburothelial injections. These authors believe that including the trigone is safe and effective without increasing the risk of vesicoureteral reflux. This will likely lead to widespread adoption of co-injection of the trigone as a standard procedure, and maybe 10-15 injections might become standard as well.

K.S. Eilber et al. (2) summarize the practical experience of a panel of experts (each with a high weekly patient throughput of 10–20 BoNT/A-DI), who discussed how and when BoNT/A-DI should be introduced to patients as an option for OAB treatment. They also discussed effective procedures for the day of treatment and follow-up care to ensure a positive treatment experience for patients and improve compliance with this long-term therapy. Important best practices include providing patients with clear advice on available OAB therapies, including BoNT/A-DI, during the initial consultation. A practice setting is preferable to an inpatient hospital setting for performing the procedure. Staff involvement (including scheduling through to follow-up treatment) is essential for a positive patient experience and compliance with this repeated therapy. The panel favored viscous lidocaine bladder instillation (diluted with 5 to 10 mL of normal saline) as an anesthetic. Room for improvement may exist to my opinion. According to the experts, up to 20 injection sites are acceptable, but fewer is preferable. Subsequent treatments should be scheduled in a standardized manner from the outset at six-month intervals, with the option of repeating the treatment earlier (at least 12 weeks) if symptoms recur. I suppose, this rigid six-month interval is likely open to discussion.

A practical discussion is presented in the article by M. Oelke (3): Is much of the BoNT/A possibly lost when injected “non-functionally” through the bladder wall into the perivesical tissue? The bladder wall becomes thinner with increasing filling and has a thickness of just 2 mm at a bladder filling of 100 mL—but only in healthy volunteers. Any injection with a longer injection needle of >2 mm and greater bladder filling than 100 mL thus appears to be ineffective into the “periphery”. The author’s main recommendations are to (1) perform pretreatment ultrasound imaging of the bladder to estimate bladder wall thickness and adjust the injection depth accordingly; (2) fill the bladder as little as possible, ideally below 100 mL(my opinion: makes it difficult to inject the flaccid bladder); (3) use short needles, ideally 2 mm; and (4) provide sufficient anesthesia and pain management to avoid patient movements during the injection therapy.

Time and again, colleagues and researchers have tried to identify predictors of good BoNT/A outcomes to inform patients about the expected success. A wide variety of literature exists on this topic, including men and women. The study presented here by M. Manso et al. (4) presents the results of 368 female patients only. The predictors of efficacy included lower pre-treatment pad usage and the absence of prior sling placement. Repeat injections (at least one) were required by 60.3% of the patients. Thus, only just over half appear to stick with the therapy or repeat it at least once. The average injection interval in this study was 18 months, due to logistical challenges (which raises the question of how patients bridge the interval when the effect of the BoNT/A-DI wears off). Of all patients, 74.5% reported a complete discontinuation of pad usage after treatment, so the rate of 100% continence is outstanding. Importantly, patients who had undergone sling placement were less likely to achieve continence. The low rate of urinary retention (1.1%) and urinary tract infections requiring antibiotics (7.9%) reflects the low-adverse-effect nature of the therapy.

A systematic review and meta-analysis by P.H. Yu et al. (5) of 26 randomized controlled trials and 3,876 patients addresses the adverse effects (especially urinary tract infections [UTIs] and urinary retention) of BoNT/A-DI in neurogenic bladder and OAB patients. The incidence of UTIs in the onabotulinumtoxinA 100 U group of OAB patients was 22.7%, compared with 9.4% in the placebo group (relative risk [RR], 2.53; *p* < 0.00001), with a trend to a higher incidence of UTI with higher BoNT/A doses. The pooled incidence of UTIs in the onabotulinumtoxinA group of neurogenic patients was 48.4% (RR, 1.54), regardless of the dosage used. The optimal regimen of antibiotic prophylaxis within the context of BoNT/A injection seems to require further studies. The most feared adverse effect among patients was urinary retention. Ten studies investigated urinary retention in the context of iOAB. The pooled retention rates were approximately 1.0% in the placebo group, 6.7% in the onabotulinumtoxinA-100U group, and 20.1% in the onabotulinumtoxinA-200U group. The risk of urinary retention was 7.32 times higher in the BoNT/A group than in the placebo group. This is a surprisingly high value, which is unlikely to be reflected in the everyday practice of high-volume centers. Again, the topic of urinary retention thus remains a topic for future research.

Our working group addressed two topics (6 and 7)—the question of pain reduction through the use of thinner needles and the patient-preferred anesthesia method: local versus general. The overall pain score for BoNT/A injections was 2.5 ± 0.3, which is in the very moderate range, and the use of thinner needles could be preferred (assuming otherwise equal quality and practicality criteria). Despite the higher anxiety and pain burden, patients still preferred local over general anesthesia overall.

A completely different use of BoNT/A injections is presented by W.C. Huang et al. (8)—not for the overactive but for the underactive bladder with chronic urinary retention, a condition that we regularly encounter in everyday practice. The treatment of this condition receives too little attention, so this article is worth reading. The authors performed a transvaginal ultrasound-guided external sphincter injection and a simultaneous transurethral bladder neck incision in 16 patients, with good therapeutic results.

Finally, H. Ibrahim et al. (9) present highly interesting and, based on previous knowledge, unexpected results. To investigate the role of SNAP-25 cleavage in the previously known BoNT/A-dependent inhibition of sensory signals, they developed a recombinant form of BoNT/A with an inactive light chain (rBoNT/A (0)), which cannot induce muscle paralysis. They also developed recombinant proteins consisting only of the light chain (LC) domain to better understand the uptake mechanisms, since the heavy chain of the protein is responsible for the internalization of the light chain. They showed that, despite lacking catalytic activity, rBoNT/A (0) inhibited the afferent signaling pathways even more strongly than catalytically active rBoNT/A. This was also evident in the studies of LC-only proteins, where the inactive rLC/A (0) protein inhibited afferent signaling pathways significantly more than the active rLC/A protein. Consequently, immunohistochemistry did not detect cleaved SNAP-25, and purinergic and nitrergic antagonists partially and completely reversed the sensory inhibition, respectively. These data suggest that BoNT/A inhibition of sensory nerve activity in this assay is not due to the classic, well-characterized “dual receptor” mechanism of BoNT/A since it is, at least partially, independent of SNAP25 cleavage and involves previously explored nitrergic and purinergic signaling mechanisms.

We hope that this Special Issue will provide you with an interesting and up-to-date compilation of important topics in urological BoNT/A therapy.

